# P-861. Incidence of Methicillin-Resistant Staphylococcus aureus (MRSA) in Sepsis of Unknown Source

**DOI:** 10.1093/ofid/ofaf695.1069

**Published:** 2026-01-11

**Authors:** Jonah J Farha, Sara Dreiling, Stephanie Harding

**Affiliations:** Oklahoma City VA Medical Center, Oklahoma City, OK; Wesley Medical Center, Wichita, Kansas; Wesley Medical Center, Wichita, Kansas

## Abstract

**Background:**

Sepsis accounts for roughly 1.7 million hospitalizations each year in the United states, a number that increased 8.6% each year over the last twenty years. The mortality rate associated with sepsis is 12.5%, and is as high as 34% in septic shock. Up to 28% of critically ill patients with sepsis or septic shock have an unknown source of infection. *Staphylococcus aureus,* particularly methicillin-resistant *S. aureus* (MRSA) is most commonly associated with mortality. Trends in MRSA rates in sepsis of unknown source are limited and are currently unknown at Wesley Medical Center. The purpose of this study is to determine the incidence of MRSA in patients presenting with sepsis of unknown source at Wesley Medical Center.

**Methods:**

This retrospective incidence study included all adult patients at Wesley Medical Center with sepsis of unknown source at time of sepsis diagnosis by provider in 2023, as identified by an ICD-10 code of A41.9. Patients with cultures positive for MRSA were evaluated for the occurrence of established MRSA risk factors, defined as history of MRSA within the last year (defined as positive MRSA culture or colonization) and/or any of the following within 90 days: hospitalization, parenteral antibiotic use, or surgery.

**Results:**

In 2023, 4,405 patients were admitted to Wesley Medical Center with sepsis of unknown source, of which 1.7% (n=77) had positive MRSA cultures. Of those 77, 62% (n=48) had at least one established risk factor. The most common risk factor was recent hospitalization, followed by parenteral antibiotic use in the last 90 days, MRSA infection in the last year, and lastly recent surgery in 90 days. 31% (n=24) patients had more than one risk factor

**Conclusion:**

The results of this study will give insight into the incidence of MRSA in patients presenting with sepsis of unknown source, a population that is underrepresented in literature. Rates of MRSA are low in patients presenting with sepsis of unknown source, and patients with positive MRSA cultures tend to have risk factors for MRSA infection.

**Disclosures:**

All Authors: No reported disclosures

